# Racial and ethnic disparities in self-reported general and mental health status among colorectal cancer survivors: impact of sociodemographic factors and implications for mortality—a SEER-CAHPS study

**DOI:** 10.1007/s11136-023-03566-z

**Published:** 2023-12-28

**Authors:** Paul S. Yoon, Stephanie Navarro, Afsaneh Barzi, Carol Y. Ochoa-Dominguez, Angel Arizpe, Albert J. Farias

**Affiliations:** 1 Department of Population and Public Health Sciences, Keck School of Medicine of USC, Los Angeles, CA USA; 2 City of Hope Comprehensive Cancer Center, Duarte, CA USA; 3 The Gehr Family Center for Health System Science, Keck School of Medicine of USC, Los Angeles, CA USA; 4 Population and Public Health Sciences, Keck School of Medicine of USC, 2001 N. Soto St., Suite 318B, Los Angeles, CA 90032 USA

**Keywords:** Colorectal cancer, Patient-reported outcomes, Racial disparities, Social risks, SEER program

## Abstract

**Purpose:**

Patient-reported outcomes are recognized as strong predictors of cancer prognosis. This study examines racial and ethnic differences in self-reported general health status (GHS) and mental health status (MHS) among patients with colorectal cancer (CRC).

**Methods:**

A retrospective analysis of Medicare beneficiaries between 1998 and 2011 with non-distant CRC who underwent curative resection and completed a Consumer Assessment of Healthcare Providers and Systems (CAHPS) survey within 6–36 months of CRC diagnosis. Analysis included a stepwise logistic regression to examine the relationship between race and ethnicity and fair or poor health status, and a proportional hazards model to determine the mortality risk associated with fair or poor health status.

**Results:**

Of 1867 patients, Non-Hispanic Black (OR 1.56, 95% CI 1.06–2.28) and Hispanic (OR 1.48, 95% CI 1.04–2.11) patients had higher unadjusted odds for fair or poor GHS compared to Non-Hispanic White patients, also Hispanic patients had higher unadjusted odds for fair or poor MHS (OR 1.92, 95% CI 1.23–3.01). These relationships persisted after adjusting for clinical factors but were attenuated after subsequently adjusting for sociodemographic factors. Compared to those reporting good to excellent health status, patients reporting fair or poor GHS or MHS had an increased mortality risk (OR 1.52, 95% CI 1.31–1.76 and OR 1.63, 95% CI 1.34–1.99, respectively).

**Conclusion:**

Racial and ethnic differences in GHS and MHS reported after CRC diagnosis are mainly driven by sociodemographic factors and reflect a higher risk of mortality. Identifying unmet biopsychosocial needs is necessary to promote equitable care.

## Introduction

Colorectal cancer (CRC) is the third most common cancer in the United States. With improvements in early detection, diagnosis, and treatment, CRC survivorship has increased significantly. More than 1.5 million CRC survivors live in the United States, with about three-fourths being 65 years or older [1]. However, advancements in CRC screening and treatment do not benefit all racial and ethnic groups equally. Differences in screening rates between non-Hispanic Black and non-Hispanic White patients account for 42% and 19% of the disparity in CRC incidence and mortality, respectively [2]. Furthermore, non-Hispanic Black, Hispanic, and non-Hispanic Asian patients are significantly less likely than non-Hispanic White patients to receive comprehensive treatment for CRC [2, 3].

These disparities may partly explain differences in CRC incidence and mortality seen across different racial and ethnic groups. For instance, non-Hispanic Black Americans display the highest CRC incidence and mortality. The incidence of CRC is 13% higher in Black Americans compared to non-Hispanic White Americans. Similarly, the mortality from CRC is 32% higher in Black Americans compared to White Americans. Hispanic and non-Hispanic Asian Americans experience lower CRC incidence and mortality compared to White Americans. However, it is probable that within both Hispanic and Asian American communities, there exist subgroups that exhibit higher incidence and mortality rates, attributed to the diverse genetic backgrounds present within these populations [4]. However, survival differences between racial and ethnic groups cannot be solely attributed to disparities in staging [5] and treatment [6]. Instead, there is growing evidence that CRC survivors continue to experience physical and psychosocial effects from their diagnosis [7].

Patient-reported outcomes (PROs) provide valuable insights into a patient’s health and healthcare experiences. These measures are especially useful for assessing quality of life and predicting CRC overall survival [8, 9]. Given CRC disparities are multifactorial, PROs may play an important role in cancer prognostics and understanding survivorship issues for racial and ethnic minorities. Self-reported general health status (GHS) and mental health status (MHS) are two single-item PRO measures that reveal differences in perceived health among racial and ethnic groups with CRC. Before CRC diagnosis, non-Hispanic Black and Hispanic patients tend to report poorer health status compared to non-Hispanic White and non-Hispanic Asian patients [10]. These differences persisted two to four years post-diagnosis, with non-Hispanic Black and Hispanic patients experiencing a worse or slower return to baseline GHS and MHS [10]. Thus, further research is necessary to identify factors driving these disparities in GHS and MHS for patients with CRC.

Cancer severity and invasive treatments may play a role in driving disparities in GHS and MHS. Patients diagnosed with more advanced CRC experience worse physical and psychological symptoms, and in turn, tend to report poorer GHS and MHS [11]. Similarly, patients treated with invasive surgeries, such as intestinal stoma construction and abdominoperineal resection, experience worse overall quality of life. Patients undergoing these procedures report physical changes in bowel, urinary, and sexual function, along with psychosocial challenges impairing body image and quality of life [12, 13]. Disparities in staging and treatment may play a role in racial and ethnic differences in PROs, and subsequently, CRC survival rates. However, there is currently no literature investigating the relationships between CRC clinical factors, PROs, and race and ethnicity.

To understand the factors behind racial and ethnic differences in GHS and MHS after CRC diagnosis, we examined whether clinical or sociodemographic factors are the main drivers. We also explored the relationship between CRC survival and immediate post-diagnosis GHS and MHS to determine whether these disparities could partly explain the observed racial and ethnic inequities in CRC survival.

## Methods

### Data source

We used the Surveillance, Epidemiology, and End Results (SEER)-Consumer Assessment of Healthcare Providers and Systems (CAHPS) linked database, which has been described in further detail elsewhere [14]. The National Cancer Institute’s SEER cancer registry provides information on patient demographics (e.g., age, gender, race and ethnicity), tumor and prognostic factors (e.g., date of diagnosis, primary tumor site, tumor morphology, stage), treatment (e.g., surgery, radiation), and outcomes (e.g., mortality, survival). The Centers for Medicare and Medicaid Services sponsored CAHPS patient experience survey collects a wide variety of patient-centered measures to assess patient experiences with their healthcare providers and quality of care.

### Study cohort

**Fig. 1:**
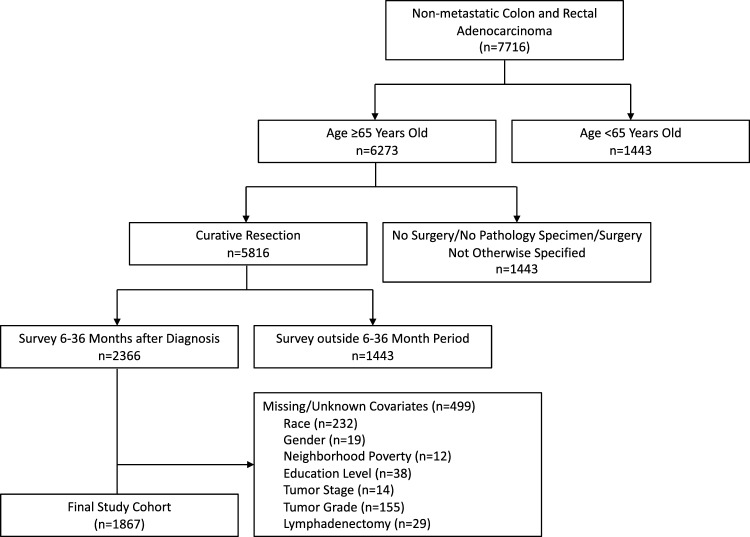
Flow chart depicting patient selection process. Inclusion criteria required patients aged 65 years or older who received curative resection for nonmetastatic colorectal cancer and completed a CAHPS survey within 6–36 months post-diagnosis. Patient with incomplete data were excluded. Evaluated variables included race, age, gender, neighborhood poverty, Medicare insurance type, education level, comorbidity count, time from diagnosis to survey, tumor characteristics (stage, grade, location), lymph node assessment, surgical approach, and radiotherapy

Our retrospective cohort included SEER-CAHPS patients who were at least 65 years old at diagnosis of nonmetastatic CRC from 1998 to 2011 who received surgical resection for their tumor and completed a CAHPS survey within 6–36 months after diagnosis. There were no cases of substantial nonresponse to CAHPS survey variables (< 2% missing), so patients were excluded from our final cohort if they were missing data for any covariates included in our analytic models (Fig. 1).

### Patient race and ethnicity

Patient race and ethnicity were extracted from self-reported CAHPS survey data using a mutually exclusive variable to categorize race and ethnicity into the following groups: non-Hispanic white, non-Hispanic black, Hispanic, and non-Hispanic Asian. Unknown (*n* = 31), other (*n* = 6), Native American (*n* = 4), and mixed (*n* = 40) racial groups were excluded from the analyses due to small sample size.

### PRO measures

Self-reported GHS and MHS are widely used and validated single-item measures to convey self-perceived health status [15, 16]. On CAHPS surveys, patients are asked, “In general, how would you rate your overall health?” and, “In general, how would you rate your overall mental or emotional health?” with response choices on a five-point Likert scale: “excellent,” “very good,” “good,” “fair,” or “poor.” We dichotomized individual responses into “fair or poor” and “excellent, very good, or good.” Previous research of CAHPS surveys demonstrated that participants reporting “excellent,” “very good,” or “good” health status were generally in good health while those reporting “fair” or “poor” were generally sicker [17]. We analyzed GHS and MHS reported within 6–36 months post-CRC diagnosis to reflect patient experiences during or shortly after the completion of cancer treatment. For patients who completed multiple surveys, the survey completed closest to the CRC diagnosis date was analyzed.

### Covariates

Covariates included in our analyses included factors that may confound the relationships between patient race and ethnicity, GHS and MHS, and mortality. These factors included sociodemographic factors (age at diagnosis, gender, percent of neighborhood living in poverty, type of Medicare insurance, highest education level completed, number of comorbidities, time between diagnosis and survey) and clinical factors (tumor stage, tumor grade, tumor location, number of lymph nodes evaluated, type of surgery, receipt of radiotherapy).

### Statistical analyses

Our analysis focused on the primary exposure of patient race and ethnicity and its relationship to self-reported health status, which served as our primary outcome of interest. We examined the distribution of sociodemographic and clinical factors by race and ethnicity and by GHS and MHS using chi-squared tests and Monte Carlo estimate if individual frequencies were less than five. To evaluate the relationship between race and ethnicity and fair or poor GHS and MHS, multivariate logistic regression models were conducted in a stepwise procedure. This involved first calculating univariate logistic regression models to test the association between race and ethnicity and GHS and MHS, followed by conducting two additional adjusted multivariate regression models: (1) adding clinical factors to race and ethnicity as covariates and then (2) adding sociodemographic factors as covariates to clinical factors and race and ethnicity. To explore the implications of GHS and MHS on patient survival, another main outcome measure of our analysis, we employed separate multivariate Cox proportional hazards models. These models adjusted for race and ethnicity along with all other covariates previously discussed. Effect modification by race and ethnicity on the association between GHS and MHS and mortality risk was assessed by the inclusion of a product interaction term between GHS and race and ethnicity as well as MHS and race and ethnicity in two additional models. These product interaction terms were found to be non-significant, so final models were run without the inclusion of product interaction terms between GHS and MHS and race and ethnicity. Statistical analyses were conducted using SAS 9.4 (SAS Institute Inc., Cary, NC, USA) with a significance level set at alpha 0.05.

## Results

### Study characteristics

Among SEER-CAHPS patients with CRC, 1,867 were diagnosed with non-distant CRC at age 65 or older, underwent surgical resection for their cancer, and completed a CAHPS survey within 6 to 36 months following CRC diagnosis. Of this cohort, 79.54% were non-Hispanic White, 6.37% were non-Hispanic Black, 7.50% were Hispanic, and 6.59% were non-Hispanic Asian (Table 1). While only 13.20% of non-Hispanic White patients and 10.57% of non-Hispanic Asian patients lived in neighborhoods with over 20% of residents living in poverty, 60.50% of non-Hispanic Black, and 34.29% of Hispanic patients lived in neighborhoods where over 20% of residents lived below the poverty line. Greater than 50% of non-Hispanic Black and Hispanic patients did not complete high school, compared to 21.95% of non-Hispanic White and 30.08% of non-Hispanic Asian patients.

**Table 1:** Characteristics of medicare beneficiaries with non-distant colorectal cancer who underwent curative resection (*N* = 1867)

	Racial and ethnic group					
	Sample *N* = 1867 (100.00)	Non-Hispanic White *N* = 1485 (79.54)	Non-Hispanic Black *N* = 119 (6.37)	Hispanic *N* = 140 (7.50)	Non-Hispanic Asian *N* = 123 (6.59)	*p*-value
Age at CRC diagnosis						0.0752
65–74 years	862 (46.17)	660 (44.44)	64 (53.78)	72 (51.43)	66 (53.66)	
75–84 years	781 (41.83)	642 (43.23)	46 (38.66)	53 (37.86)	40 (32.52)	
85 + years	224 (12.00)	183 (12.32)	9 (7.56)	15 (10.71)	17 (13.82)	
Gender						0.0005
Male	876 (46.92)	708 (47.68)	38 (31.93)	79 (56.43)	51 (41.46)	
Female	991 (53.08)	777 (52.32)	81 (68.07)	61 (43.57)	72 (58.54)	
Percent of residents in neighborhood living in poverty						< 0.0001
< 5%	490 (26.25)	431 (29.02)	5 (4.20)	19 (13.57)	35 (28.46)	
5% to < 10%	519 (27.80)	432 (29.09)	10 (8.40)	28 (20.00)	49 (39.84)	
10% to < 20%	529 (28.33)	426 (28.69)	32 (26.89)	45 (32.14)	26 (21.14)	
20% to 100%	329 (17.62)	196 (13.20)	72 (60.50)	48 (34.29)	13 (10.57)	
Medicare insurance type						< 0.0001
Medicare advantage	1067 (57.15)	810 (54.55)	75 (63.03)	98 (70.00)	84 (68.29)	
Fee for service	800 (42.85)	675 (45.45)	44 (36.97)	42 (30.00)	39 (31.71)	
Education level						< 0.0001
Less than high school	504 (27.00)	326 (21.95)	64 (53.78)	77 (55.00)	37 (30.08)	
High school	640 (34.28)	538 (36.23)	31 (26.05)	34 (24.29)	37 (30.08)	
High school or greater	723 (38.73)	621 (41.82)	24 (20.17)	29 (20.71)	49 (39.84)	
Number of comorbidities						0.0216
No comorbidities	1367 (73.22)	1105 (74.41)	72 (60.50)	102 (72.86)	88 (71.54)	
1 Comorbidity	338 (18.10)	255 (17.17)	29 (24.37)	26 (18.57)	28 (22.76)	
2 + Comorbidities	162 (8.68)	125 (8.42)	18 (15.13)	12 (8.57)	7 (5.69)	
Diagnosis to survey duration						0.3831
6–12 Months	481 (25.76)	380 (25.59)	37 (31.09)	33 (23.57)	31 (25.20)	
12–24 Months	758 (40.60)	619 (41.68)	38 (31.93)	54 (38.57)	47 (38.21)	
24–36 Months	628 (33.64)	486 (32.73)	44 (36.97)	53 (37.86)	45 (36.59)	
Tumor stage						0.0239
In Situ/localized	1062 (56.88)	863 (58.11)	70 (58.82)	74 (52.86)	55 (44.72)	
Regional	805 (43.12)	622 (41.89)	49 (41.18)	66 (47.14)	68 (55.28)	
Tumor grade						0.0798
Well differentiated	220 (11.78)	179 (12.05)	17 (14.29)	14 (10.00)	10 (8.13)	
Moderately differentiated	1347 (72.15)	1050 (70.71)	90 (75.63)	108 (77.14)	99 (80.49)	
Poorly differentiated/undifferentiated	300 (16.07)	256 (17.24)	12 (10.08)	18 (12.86)	14 (11.38)	
Tumor location						0.0038
Rectal	435 (23.30)	360 (24.24)	14 (12.76)	30 (21.43)	31 (25.20)	
Right-sided colon	919 (49.22)	734 (49.43)	72 (60.50)	66 (47.14)	47 (38.21)	
Left-sided colon	513 (27.48)	391 (26.33)	33 (27.73)	45 (31.43)	45 (36.59)	
Number of lymph nodes evaluated						0.5376
< 12 nodes evaluated	833 (44.62)	659 (44.38)	53 (44.54)	70 (50.00)	51 (41.46)	
≥ 12 nodes evaluated	1034 (55.38)	826 (55.62)	66 (55.46)	70 (50.00)	72 (58.54)	
Surgical approach						0.0687
Subtotal resection	1753 (93.89)	1384 (93.20)	116 (97.48)	133 (95.00)	120 (97.56)	
Total resection	114 (6.11)	101 (6.80)	3 (2.52)	7 (5.00)	3 (2.44)	
Received radiotherapy						0.0499
No	1690 (90.52)	1343 (90.44)	114 (95.80)	128 (91.43)	105 (85.37)	
Yes	177 (9.48)	142 (9.56)	5 (4.20)	12 (8.57)	18 (14.64)	

Findings reported as N (%) and *p-*values reported using Chi-square tests or the Monte Carlo estimate of the exact test when individual cell counts were less than 5

Regarding PRO measures, 33.86% of non-Hispanic White patients and 34.71% of non-Hispanic Asian patients reported fair or poor GHS versus 44.35% of non-Hispanic Black patients and 43.17% of Hispanic patients (Table 2). In addition, the proportion of patients reporting fair or poor GHS increased with age, the percent living in poverty in one’s neighborhood, decreasing education level, and increasing number of comorbidities. Hispanic patients reported the highest proportion of fair or poor MHS, at 20.59%. The proportion of patients reporting fair or poor MHS increased with age and decreasing education level. Fair or poor MHS was greatest among patients living in neighborhoods with over 20% of residents living in poverty.

**Table 2:** Distribution of fair or poor self-reported general and mental health status among Medicare beneficiaries with non-distant colorectal cancer

	Fair or poor general health status	Sample *N* = 1825	*p*-value	Fair or poor mental health status	Sample *N* = 1782	*p*-value
Race and ethnicity			0.0265			0.0331
Non-Hispanic white	491 (33.86)	1450		168 (11.87)	1415	
Non-Hispanic black	51 (44.35)	115		16 (14.16)	113	
Hispanic	60 (43.17)	139		28 (20.59)	136	
Non-Hispanic Asian	42 (34.71)	121		16 (13.56)	118	
Age at CRC diagnosis			0.0242			< 0.001
65–74 years	273 (32.16)	849		75 (9.08)	826	
75–84 years	284 (37.37)	760		116 (15.47)	750	
85 + years	87 (40.28)	216		37 (17.96)	206	
Gender			0.3468			0.6474
Male	312 (36.41)	857		104 (12.41)	838	
Female	332 (34.30)	968		124 (13.14)	944	
Percent of residents in neighborhood living in poverty			0.0040			0.0044
< 5%	144 (30.00)	480		42 (9.11)	461	
5% to < 10%	170 (33.73)	504		64 (12.96)	494	
10% to < 20%	196 (37.84)	518		65 (12.77)	509	
20% to 100%	134 (41.49)	323		57 (17.92)	318	
Medicare insurance type			0.0751			0.1552
Medicare advantage	349 (33.56)	1040		118 (11.80)	1000	
Fee for service	295 (37.58)	785		110 (14.07)	782	
Education level			< 0.0001			< 0.0001
Less than high school	231 (47.05)	491		99 (20.93)	473	
High school	211 (33.71)	626		74 (12.03)	615	
High school or greater	202 (28.53)	708		55 (7.93)	694	
Number of comorbidities			< 0.0001			0.0131
No comorbidities	438 (32.74)	1338		169 (12.92)	1308	
1 Comorbidity	112 (34.36)	326		29 (9.24)	314	
2 + Comorbidities	94 (58.39)	161		30 (18.75)	160	
Diagnosis to survey duration			0.4100			0.4388
6–12 Months	174 (37.58)	463		48 (11.29)	425	
12–24 Months	263 (35.21)	747		94 (12.67)	742	
24–36 Months	207 (33.66)	615		86 (18.98)	615	
Tumor stage			0.2243			0.5660
In Situ/localized	354 (34.10)	1038		134 (13.19)	1016	
Regional	290 (36.85)	787		94 (12.27)	766	
Tumor grade			0.4788			0.6259
Well differentiated	74 (34.42)	215		26 (12.44)	209	
Moderately differentiated	459 (34.75)	1321		171 (13.21)	1294	
Poorly differentiated/undifferentiated	111 (38.41)	289		31 (11.11)	279	
Tumor location			0.0389			0.7056
Rectal	166 (39.34)	422		51 (12.53)	407	
Right-sided colon	319 (35.60)	896		118 (13.42)	879	
Left-sided colon	159 (31.69)	507		59 (11.90)	496	
Number of lymph nodes evaluated			0.0018			0.0757
< 12 nodes evaluated	319 (39.19)	814		113 (14.38)	786	
≥ 12 nodes evaluated	325 (32.15)	1011		115 (11.44)	996	
Surgical approach			0.0439			0.1347
Subtotal resection	595 (34.71)	1714		209 (12.49)	1673	
Total resection	49 (44.14)	111		19 (17.43)	109	
Received radiotherapy			0.0961			0.1915
No	573 (34.69)	1652		212 (13.13)	1615	
Yes	71 (41.04)	173		16 (9.58)	167	

### Drivers of fair or poor health status

Compared to non-Hispanic White patients, unadjusted results showed that non-Hispanic Black patients were 56% more likely (OR 1.56, 95% CI 1.06–2.28) and Hispanic patients were 48% more likely (OR 1.48, 95% CI 1.04–2.11) to report fair or poor GHS (Table 3). These findings persisted after controlling for potential confounding factors that were related to clinical characteristics, including tumor stage, tumor grade, tumor location, the number of lymph nodes evaluated at lymphadenectomy, the surgical approach, and receipt of radiotherapy. Patient race and ethnicity were significantly associated with odds of reporting fair or poor GHS in the unadjusted analysis (*p* = 0.0276) and after controlling for clinical factors (*p* = 0.0189). However, after additionally controlling for potential confounding factors relating to sociodemographic characteristics, there were no significant associations between patient race and ethnicity and odds of reporting fair or poor GHS (*p* = 0.5251).

**Table 3:** Multivariable logistic regression analyses of associations between race and ethnicity and self-reported general and mental health status among Medicare beneficiaries with non-distant colorectal cancer

	Fair or poor general health status OR (95% CI)	*p*-value	Fair or poor mental health status OR (95% CI)	*p*-value
Model 1 (Race and ethnicity)				
^a^Race and ethnicity		0.0276		0.0367
Non-Hispanic White	1.00 (Ref)		1.0 (Ref)	
Non-Hispanic Black	1.56 (1.06, 2.28)		1.22 (0.70, 2.13)	
Hispanic	1.48 (1.04, 2.11)		1.92 (1.23, 3.01)	
Non-Hispanic Asian	1.04 (0.70, 1.53)		1.16 (0.67, 2.02)	
Model 2 (Race and ethnicity + clinical)				
^b^Race and ethnicity		0.0189		0.0391
Non-Hispanic White	1.0 (Ref)		1.0 (Ref)	
Non-Hispanic Black	1.62 (1.10, 2.40)		1.21 (0.69, 2.11)	
Hispanic	1.49 (1.04, 2.13)		1.92 (1.22, 3.00)	
Non-Hispanic Asian	1.08 (0.72, 1.60)		1.25 (0.71, 2.18)	
Model 3 (Race and ethnicity + clinical + sociodemographic)				
^c^Race and ethnicity		0.5251		0.2254
Non-Hispanic White	1.0 (Ref)		1.0 (Ref)	
Non-Hispanic Black	1.25 (0.81, 1.92)		0.83 (0.45, 1.53)	
Hispanic	1.25 (0.86, 1.82)		1.51 (0.93, 2.45)	
Non-Hispanic Asian	1.14 (0.75, 1.71)		1.33 (0.75, 2.37)	

Findings reported as odd ratio (95% confidence interval)

^a^Unadjusted analysis

^b^Analysis adjusted for: tumor stage, tumor grade, tumor location, number of lymph nodes evaluated, surgical approach, and radiotherapy

^c^Analysis adjusted for: tumor stage, tumor grade, tumor location, number of lymph nodes evaluated, surgical approach, radiotherapy, age at CRC diagnosis, gender, percent of residents in neighborhood living in poverty, Medicare insurance type, education level, number of comorbidities, and diagnosis to survey duration

Regarding MHS, unadjusted results showed that Hispanic patients were 92% more likely to report fair or poor MHS compared to non-Hispanic White patients (OR 1.92, 95% CI 1.23–3.01) (Table 3). This finding also persisted after controlling for clinically related factors. Both the unadjusted analysis (*p* = 0.0367) and analysis controlling for clinical factors (*p* = 0.0391) demonstrated significant associations between patient race and ethnicity and odds of reporting fair or poor MHS. However, this association did not maintain statistical significance after additionally controlling for sociodemographic factors (*p* = 0.2254).

### Mortality and fair or poor health status

The median follow-up time for CRC patients in our study was 5.75 years. In fully adjusted models, reporting fair or poor GHS was associated with a 52% increased risk of mortality (HR 1.52, 95% CI 1.31–1.76), and reporting fair or poor MHS was associated with a 63% increased risk of mortality (HR 1.63, 95% CI 1.34–1.99) (Table 4). Additional factors associated with increased mortality risk included older age and having regional stage cancer. Factors associated with decreased mortality risk included being non-Hispanic Asian, being female, graduating from high school or greater, and receiving radiotherapy.

**Table 4:** Multivariable Cox proportional hazards regression analyses of associations between self-reported general and mental health status with survival among Medicare beneficiaries with non-distant colorectal cancer

Variables	Self-reported health status up to 36 months from colorectal cancer diagnosis			
	Fair or poor general health status HR (95% CI)	*p*-value	Fair or poor mental health status HR (95% CI)	*p*-value
Fair or poor general health status^*^	1.52 (1.31, 1.76)	< 0.0001	N/A	
Fair or poor mental health status^*^	N/A		1.63 (1.34, 1.99)	< 0.0001
Race/ethnicity		0.0217		0.0210
Non-Hispanic White	1.0 (Ref)		1.0 (Ref)	
Non-Hispanic Black	0.83 (0.59, 1.15)		0.86 (0.62, 1.21)	
Hispanic	0.83 (0.63, 1.09)		0.78 (0.59, 1.04)	
Non-Hispanic Asian	0.61 (0.43, 0.86)		0.61 (0.43, 0.87)	
Age at survey		< 0.0001		< .0001
65–74 years	1.0 (Ref)		1.0 (Ref)	
75–84 years	1.72 (1.47, 2.02)		1.73 (1.47, 2.04)	
85 + years	3.10 (2.50, 3.83)		3.17 (2.54, 3.96)	
Gender		0.0003		0.0011
Male	1.0 (Ref)		1.0 (Ref)	
Female	0.77 (0.66, 0.89)		0.78 (0.67, 0.91)	
Neighborhood poverty level		0.2858		0.2866
< 5%	1.0 (Ref)		1.0 (Ref)	
5 to < 10%	1.21 (0.998, 1.48)		1.22 (0.99, 1.49)	
10 to < 20%	1.13 (0.93, 1.37)		1.16 (0.95, 1.42)	
20 to 100%	1.14 (0.90, 1.45)		1.14 (0.90, 1.46)	
Medicare insurance type		0.6536		0.3163
Medicare advantage	1.0 (Ref)		1.0 (Ref)	
Fee for service	1.03 (0.90, 1.19)		1.08 (0.93, 1.25)	
Education level		0.0015		0.0011
Less than high school	1.0 (Ref)		1.0 (Ref)	
High school	0.90 (0.75, 1.07)		0.91 (0.76, 1.09)	
High school or greater	0.72 (0.60, 0.86)		0.71 (0.59, 0.86)	
Number of comorbidities		0.7866		0.6295
No comorbidities	1.0 (Ref)		1.0 (Ref)	
1 Comorbidity	0.94 (0.73, 1.19)		0.95 (0.73, 1.23)	
2 + Comorbidities	1.06 (0.77, 1.46)		1.14 (0.83, 1.58)	
Diagnosis to survey duration		0.0259		0.0351
6–12 Months	1.0 (Ref)		1.0 (Ref)	
12–24 Months	0.82 (0.68, 0.97)		0.83 (0.69, 0.995)	
24–36 Months	0.79 (0.66, 0.95)		0.78 (0.65, 0.95)	
Tumor stage		0.0002		< 0.0001
In Situ/localized	1.0 (Ref)		1.0 (Ref)	
Regional	1.33 (1.14, 1.54)		1.36 (1.17, 1.58)	
Tumor grade		0.5752		0.4229
Well differentiated	1.0 (Ref)		1.0 (Ref)	
Moderately differentiated	1.13 (0.90, 1.42)		1.17 (0.93, 1.48)	
Poorly differentiated/undifferentiated	1.13 (0.85, 1.50)		1.16 (0.87, 1.55)	
Tumor location		0.6215		0.6657
Rectal	1.0 (Ref)		1.0 (Ref)	
Right-sided colon	0.90 (0.73, 1.11)		0.91 (0.73, 1.13)	
Left-sided colon	0.93 (0.74, 1.16)		0.92 (0.73, 1.15)	
Lymphadenectomy		0.1830		0.0514
< 12 nodes evaluated	1.0 (Ref)		1.0 (Ref)	
≥ 12 nodes evaluated	0.90 (0.78, 1.05)		0.86 (0.74, 1.00)	
Surgical approach		0.0605		0.0556
Subtotal resection	1.0 (Ref)		1.0 (Ref)	
Total resection	1.32 (0.99, 1.77)		1.33 (0.99, 1.79)	
Received radiotherapy		0.0245		0.0204
No	1.0 (Ref)		1.0 (Ref)	
Yes	0.71 (0.53, 0.96)		0.70 (0.51, 0.95)	

Findings reported as hazard ratio (95% confidence interval)

^*^Reference = Good, Very Good, or Excellent Health Status

## Discussion

We examined GHS and MHS from a large nationally representative cohort in the SEER-CAHPS dataset, specifically during the period immediately following diagnosis of CRC, to identify clinical and sociodemographic factors driving racial and ethnic disparities in PROs. Our findings suggest clinical practices that address a patient’s social needs may be most effective in reducing these disparities during the immediate post-CRC diagnosis period. Additionally, we observed that patients who reported fair or poor GHS and MHS during this period had a higher risk of mortality.

### Disparities in patient-reported outcomes

In our study, non-Hispanic Black and Hispanic patients had significantly higher odds of reporting fair or poor GHS and MHS compared to non-Hispanic White patients. Specifically, Black and Hispanic patients were 56% and 48% more likely, respectively, to report poorer GHS, and Hispanic patients were 92% more likely to report poorer MHS compared. Similarly, prior research has demonstrated racial and ethnic disparities in PROs in the context of CRC diagnosis. One study demonstrated that Hispanic patients experienced a more significant increase in poor MHS after CRC diagnosis compared to other racial groups [10]. The reason for this sharp decline in MHS among Hispanic patients is not evident in the study; however, the financial impact of cancer, potentially exacerbated by a higher number of Hispanics living in impoverished areas, is suggested as a potential explanation. Thus, efforts to further understand race and ethnic-specific relationships between CRC diagnosis and PROs are necessary for targeted interventions and equitable care.

While prior research has demonstrated significant racial and ethnic disparities in PROs following CRC diagnosis, it is important to recognize the impact of sociodemographic factors on the relationship between race and ethnicity and PROs. After controlling for all sociodemographic factors collectively (subsequent to controlling for clinical factors), associations between patient race and ethnicity and GHS and MHS were attenuated, suggesting that sociodemographic characteristics mainly drive racial and ethnic disparities in PROs. These sociodemographic factors included age at CRC diagnosis, gender, neighborhood poverty concentration, Medicare insurance type, education level, number of comorbidities, and time elapsed from diagnosis to CAHPS survey completion. Previous research has also identified gender, education, financial dependence, and life stress as major contributors to disparities in self-reported health [18–21]. Meraya et al. found that increased labor income was consistently associated with higher self-reported health, regardless of patient race and ethnicity, suggesting that financial independence and autonomy may be a more influential factor of GHS and MHS than patient race and ethnicity [22].

Coughlin et al. demonstrated that among cancer survivors, low-income patients experienced over two times the odds of reporting fair or poor health compared to higher income patients [23]. In another study of 1785 patients with CRC, researchers found that the strongest predictors of a positive depression screen among elderly CRC survivors were not tumor-specific factors, but lower socioeconomic status, higher number of comorbidities, and impaired preoperative activities of daily living [24]. Other studies have also highlighted financial toxicity as a significant barrier to quality cancer care and health status, with financial burden being associated with lower treatment adherence, resulting in a higher risk of recurrence and shorter survival. Patients may also face financial and emotional distress having to prioritize paying for treatment over essential household needs like food, shelter, and clothing. Conversely, patients who are educated about the cost of cancer treatment can make informed decisions that improve their outcomes [25, 26]. These findings underscore the importance of community-level educational outreach and financial assistance as preventative measures before cancer diagnosis. From a policy- and decision-maker perspective, upstream interventions that offer financial support could improve PROs.

According to our study, racial and ethnic differences in tumor burden and cancer treatment do not appear to drive disparities in PROs among patients with CRC. However, our study excluded patients with metastatic disease, and there is strong evidence that increasing cancer severity is associated with decreased quality of life. In Funk et al., patients with head and neck cancer reported decreased GHS with increased cancer stage [27]. Likewise, in Hung et al., patients with stage IV CRC undergoing palliative treatment reported the worst quality of life measures, which are PROs with similar utility as GHS and MHS [11]. However, in regards to clinical factors, studies have consistently shown that both patient quality of life and GHS before and after cancer treatment have similar scores, suggesting that cancer treatment alone cannot fully explain the potential variations in self-reported physical and psychosocial scores [27–30]. Although clinical factors may play a role in determining GHS and MHS among patients with cancer, in our cohort, this role does not appear to be more influential than patient race and ethnicity. Social risks, such as housing and food insecurity, create barriers to quality cancer care and contribute to poorer outcomes, especially for marginalized communities. Structural interventions targeting social needs, such as education, income, and healthcare access, may have a more significant impact in addressing racial and ethnic differences in PROs reported around the time of CRC diagnosis and first course of treatment. Directing resources towards downstream medical interventions post-diagnosis may yield only mild benefits towards PROs and subsequently mortality risk.

### Patient-reported outcomes and CRC survival

Fair or poor GHS and MHS shortly after CRC diagnosis were associated with over 50% and 60% increased risk of mortality, respectively, in our cohort. Similarly, Chavan et al. found that poor self-reported health was associated with about 2.8 times the risk of three-year mortality in a cohort of older adult cancer survivors [31]. Functional limitation, a PRO similar to GHS and MHS, was also associated with close to 20% increased risk of mortality [32]. In fact, several studies have shown that PROs can be more sensitive prognostic indicators than physician reported outcomes among patients with cancer [33–35]. To our knowledge, this is the first study to investigate racial and ethnic disparities in PROs and their association with CRC mortality. A study of lung cancer patients demonstrated variations in patient experiences with care based on their racial and ethnic backgrounds. When the study explored the impact of these patient experiences on mortality, it found that better patient-reported experiences with specialist physicians were associated with decreased mortality risk among White, Hispanic, and Asian patients diagnosed with lung cancer [36]. These findings highlight the intricate relationship between patient experiences with care and their outcomes, underscoring the need to address racial and ethnic disparities in healthcare to improve patient well-being.

### Study strengths and limitations

Our study, based on a nationally representative and population-based dataset, provided a relatively large sample of patients with CRC who completed a CAHPS survey. However, due to smaller sample sizes of racial and ethnic groups and a large number of predictors, the study's power may be reduced. Despite CRC treatment typically being completed within the first year after diagnosis, we included patient surveys up to 36 months post-diagnosis to ensure an adequate sample size. Conducting sensitivity analysis, we found no significant difference in PROs between the 12-month, 24-month, and 36-month time points. The use of SEER-CAHPS data allowed for reliable and comprehensive measures of individual sociodemographic and clinical factors as potential confounding variables in the relationships between race and ethnicity, PROs, and mortality. However, as with all observational studies, there is a risk of residual confounding, and we were unable to control for certain clinical factors like date of treatment, receipt of chemotherapy, or receipt of colostomy; as well as certain sociodemographic factors like geographic proximity to treating healthcare center and individual income level, which may also act as potential confounding variables.

Our study population was limited to Medicare enrollees aged 65 and older, limiting generalizability to younger age groups and the uninsured. However, it is important to recognize the unique challenges and characteristics of the older adult population in the context of cancer and chronic diseases. Older adults, despite constituting the majority of cancer patients, are frequently underrepresented in clinical trials, especially those with multiple comorbidities. Also, clinical trials tend to prioritize cancer-specific endpoints, such as progression-free and overall survival, potentially failing to capture outcomes that matter most to older patients, such as changes in physical and social functioning, which significantly affect their quality of life [37]. These observations align with a separate study involving 115 older adults aged 65 or older, where over half of the participants exhibited a substantial disease burden according to the Charlson Comorbidity Index score. The findings from this study underscore how the high prevalence of multiple chronic diseases among older adults compromises their quality of life in both physical and mental domains [38].

Furthermore, use of self-reported PROs and additional variables from CAHPS survey data, as well as a complete case analysis approach, excluded some potentially eligible patients due to nonresponse to specific CAHPS survey questions. Nonetheless, nonresponse rates to individual CAHPS questions included in our research were generally low, with the highest rate of unintended and intended nonresponse being about 2.2% and 2.3%, respectively, of respondents to the MHS question. Proxy reports of GHS and MHS represented 16.7% of our cohort and were also included in our study. Previous studies demonstrated that proxy-reported scores were significantly lower than patient scores. In general, these studies suggest a stronger agreement between proxy-patient scores in health domains that are easier to observe, such as physical function, in comparison to less visible domains like emotional function. However, in our cohort, a higher proportion of patients who reported fair or poor GHS and MHS, are non-Hispanic Black and Hispanic, live in poorer neighborhoods, and had lower educational attainment utilized a proxy. Since these subgroups are populations of interest in our study, exclusion of proxy-reported scores could lead to biased estimates.

## Conclusion

In summary, our study shows racial and ethnic disparities in PROs reported after CRC diagnosis, with non-Hispanic Black and Hispanic patients reporting poorer health status than non-Hispanic White patients. These disparities are mainly driven by sociodemographic factors rather than clinical factors. Our study demonstrated the relative weight of sociodemographic factors over clinical factors but was not able to identify specific factors that hold the most explanatory power. Future research may explore these factors to suggest specific macro-level structural policies or patient-provider interactions aimed at improving PROs. Our study also found that fair or poor GHS and MHS after CRC diagnosis were associated with higher mortality risk. These findings highlight the need to address unmet sociodemographic barriers to optimal health (such as limited financial independence, limited education, and increased life stress), especially among non-Hispanic Black and Hispanic patients. While our study underscores the elevated mortality risk associated with poorer self-reported health status, further research could employ mediation analysis to pinpoint the specific factors contributing to these disparities. To reduce disparities in cancer outcomes and promote equitable care, health care organizations will need to increasingly implement approaches to address social risks across the cancer care continuum.
